# Efflux pump proteins in antifungal resistance

**DOI:** 10.3389/fphar.2014.00202

**Published:** 2014-08-29

**Authors:** Rajendra Prasad, Manpreet K. Rawal

**Affiliations:** Membrane Biology Laboratory, School of Life Sciences, Jawaharlal Nehru UniversityNew Delhi, India

**Keywords:** multidrug resistance, ABC transporters, MFS transporters, azoles, efflux pumps, *Candida*

## Abstract

It is now well-known that the enhanced expression of ATP binding cassette (ABC) and major facilitator superfamily (MFS) proteins contribute to the development of tolerance to antifungals in yeasts. For example, the azole resistant clinical isolates of the opportunistic human fungal pathogen *Candida albicans* show an overexpression of Cdr1p and/or CaMdr1p belonging to ABC and MFS superfamilies, respectively. Hence, azole resistant isolates display reduced accumulation of therapeutic drug due to its rapid extrusion and that facilitates its survival. Considering the importance of major antifungal transporters, the focus of recent research has been to understand the structure and function of these proteins to design inhibitors/modulators to block the pump protein activity so that the drug already in use could again sensitize resistant yeast cells. The review focuses on the structure and function of ABC and MFS transporters of *Candida* to highlight the recent advancement in the field.

## Introduction

The occurrence of fungal infections has risen dramatically over the past few decades because of the increase in number of immunocompromised patients undergoing, transplantation surgery, cancer chemotherapy and having an HIV infection etc. (Richardson, 2005). The severity of fungal infections varies from superficial to life-threatening systemic infections. When fungi penetrate the epithelial surfaces of immunocompromised hosts they cause invasive fungal infections that are associated with high morbidity and mortality. The fungal genera most often associated with invasive fungal infections include *Candida, Aspergillus and Cryptococcus* (Pfaller and Diekema, 2007). The opportunistic *C. albicans* accounts for approximately 50–60% causes of candidiasis particularly in immunocompromised patients. Superficial infections caused by *C. albicans* are commonly treated with azole drugs while life-threatening systemic infections are treated with triazole drugs, or the more recent and expensive echinocandins (Perlin, 2011).

**Figure 1:**
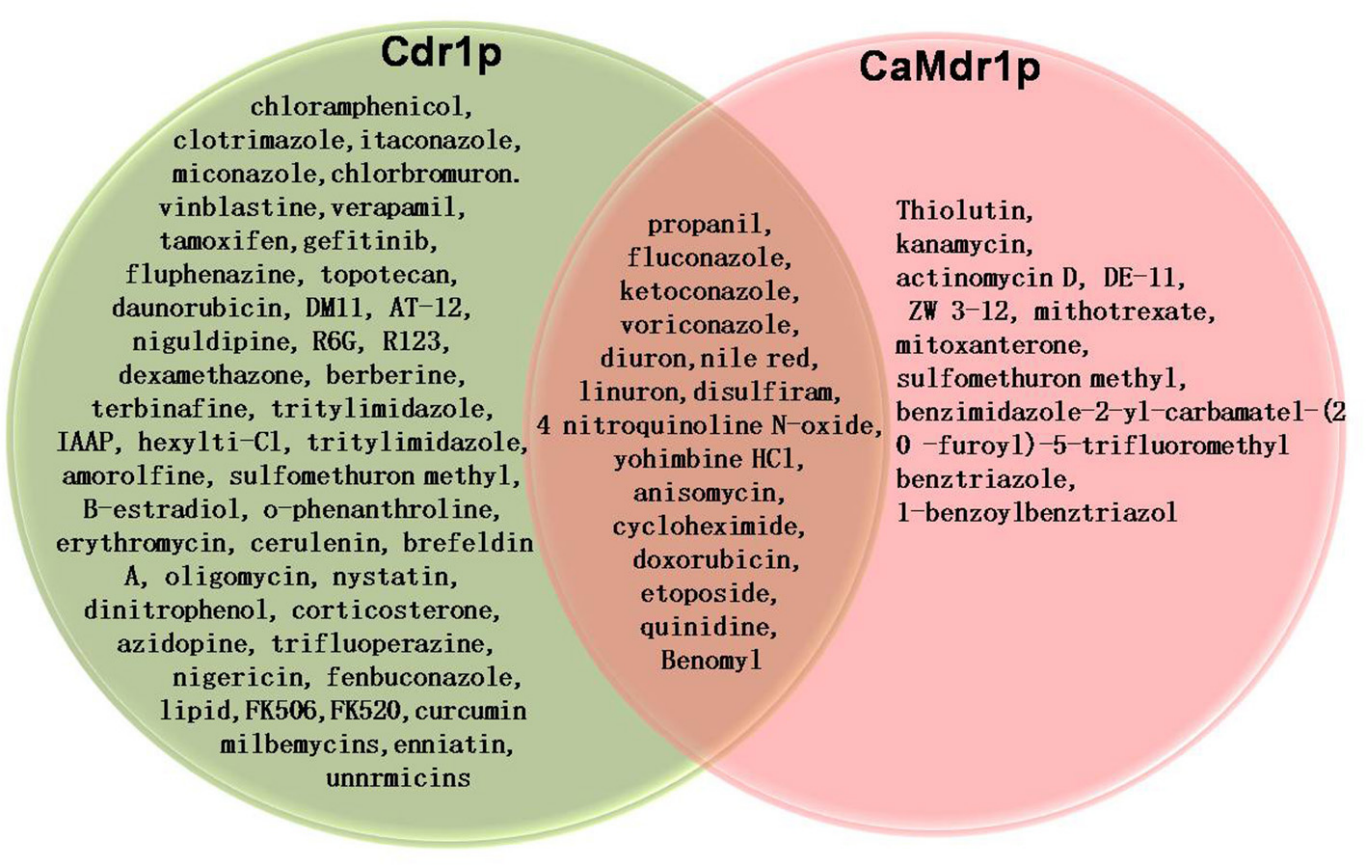
**Venn diagram showing substrates which are common and distinct for Cdr1p and CaMdr1p**.

The widespread and prolonged use of antifungals results not only in the development of tolerance toward the not only drug in use, but also in the development of collateral resistance to other drugs and to a variety of unrelated compounds (Figure 1). The development of resistance to a variety of unrelated compounds is termed as multidrug resistance (MDR). The clinical emergence of MDR is common occurrence which poses a major hurdle in antifungal therapy. Notably, MDR is not restricted to fungi but it is a wide occurrence phenomena observed in various organisms throughout the evolutionary scale. Combating MDR poses major challenge to clinicians since it is also a multi-factorial phenomenon where a combination of mechanisms could contribute in the development of drug tolerance. The several mechanisms of MDR which have been characterized in yeast, includes the development of *Candida* cell's inability to accumulate drugs intracellularly to toxic levels due to an overexpression of membrane-associated transporters acting as multidrug efflux pumps. The rapid efflux in resistant strains ensures that the drug is not accumulated to lethal levels. Several azole-resistant clinical isolates of *C. albicans* as well as of other fungal pathogens like *Aspergillus fumigates* and *Cryptococcus neoformans* display transcriptional activation of efflux pump encoding genes and often show reduced intracellular accumulation of drugs, thus confirming the role of efflux proteins in drug extrusion and tolerance (Prasad et al., 2002).

There are two main classes of transport proteins which are mainly responsible for the development of antifungal resistance, involving different mechanistic strategies to efflux drugs. For example, while superfamily of ATP-binding cassette (ABC) proteins are primary active transporters that employ energy from the hydrolysis of ATP to drive the efflux of drugs, those belonging to major facilitator superfamily (MFS) are secondary active transporters that utilize the electrochemical gradient of protons across the plasma membrane to efflux substrates (Cannon et al., 2009). Both classes of pumps are integral membrane proteins with distinctive functional domains. Because of the importance of drug transporters in MDR, there has been a spurt in research on all aspects of these transporters. In this review, we update studies on the structure and function of these transporters particularly those belonging to *C. albicans*.

## ABC proteins in *Candida*

The release of the genome sequence of the diploid human pathogenic fungus *C. albicans* made it possible to analyse the superfamily of ABC proteins (Braun et al., 2005). The genome wide inventory of *C. albicans* reveals that it has 28 putative ABC proteins including 12 half transporters that remain uncharacterized (Gaur et al., 2005). By employing neighbor-joining tree and self-organizing-map-based clustering methods, these 28 putative ABC proteins can be grouped into five known subfamilies: PDR (pleiotropic drug resistance), MDR (multidrug resistance), MRP (multidrug resistance-associated protein), RLI (RNase L inhibitor)/ALDP (adrenoleukodystrophy protein), and YEF3 (yeast elongation factor EF-3), and a sixth “others” category that includes soluble (Table 1) ABC non-transporter proteins unrelated to the existing fungal subfamilies. The PDR protein subfamily of *C. albicans* comprises seven full-sized members: Cdr1p (Prasad et al., 1995), Cdr2p (Sanglard et al., 1997), Cdr3p (Balan et al., 1997), Cdr4p (Franz et al., 1998), Cdr11p (Ca918, assembly #20 http://www.candidagenome.org/download/Assembly20notes/), CaSnq2p, and Ca4531 (Gaur et al., 2005). However, only two proteins of PDR subfamily viz Cdr1p and Cdr2p have been shown to be multidrug transporters (Prasad et al., 1995; Sanglard et al., 1997). The other well characterized close homologs such as Cdr3p and Cdr4p are not drug transporters but are involved in phospholipids translocation within the lipid bilayer of natural membrane.

**Table 1:** **ABC transporters of *Candida albicans***.

**Subfamily^a^**	**ORF names**	**Gene name**	**Size^b^**	**Location**	**Topology^c^**	**Function**	**References**
PDR	CaO19.6000	CaCDR1	1501	PM	(NBD-TMS6)_2_	Drug effIux, phospholipid translocation	Prasad et al., 1995
	CaO19.5958	CaCDR2	1499	PM	(NBD-TMS6)_2_	Drug effIux, phospholipid translocation	Sanglard et al., 1997
	CaOI9.1312	CaCDR3	1501	PM	(NBD-TMS6)_2_	Opaque-phase specific transporter, phospholipid translocation	Balan et al., 1997; Smriti et al., 2002
	CaO19.5079	CaCDR4	1490	ND	(NBD-TMS6)_2_	Phospholipid translocation	Franz et al., 1998
	CaO19.918	CaCDR99	683	ND	NBD-TMS6	Drug efflux pump	Gaur et al., 2005
	CaO19.919		819	ND	NBD-TMS6	ND	Gaur et al., 2005
	CaO19.5759		1493	ND	(NBD-TMS6)_2_	ND	Gaur et al., 2005
	CaO19.4531		1245	ND	(NBD-TMS6)_2_	ND	Gaur et al., 2005
	CaO19.3120		584	ND	NBD-TMS6	ND	Gaur et al., 2005
	CaO19.459		719	ND	TMS1-NBD-TMS7	ND	Gaur et al., 2005
MDR	CaO19.7440	CaHST6	1328	ND	(TMS6-NBD)_2_	Transport of α-factor, drugs	Raymond et al., 1998
	CaO19.2615	CaMDL1	659	ND	TMS6-NBD	ND	Gaur et al., 2005
	CaOI9.13043		726	ND	TMS6-NBD	ND	Gaur et al., 2005
	CaOI9.1077		730	ND	TMS6-NBD	ND	Gaur et al., 2005
MRP/CFTR	CaO19.6478	CaYOR1	711	PM	TMS6-NBD	Drug efflux pump	Gaur et al., 2005
	CaO19.1783	CaYCF1	1512	Vacoule	(TMS6-NBD)_2_	Drug efflux pump	Gaur et al., 2005
	CaO19.5100	CaMLT1	1544	Vacoule	(TMS6-NBD)_2_	Transport involved in virulence	Theiss et al., 2002
	CaO19.6382		1254	ND	(TMS6-NBD)_2_	ND	Gaur et al., 2005
	CaO19.1784		763	ND	TMS6-NBD	ND	Gaur et al., 2005
ALDp	CaO19.7500		652	ND	TMS6-NBD	ND	Gaur et al., 2005
	CaO19.5255		615	ND	TMS6-NBD	ND	Gaur et al., 2005
EF3	CaO19.6060		751	ND	NBD-NBD	ND	Gaur et al., 2005
	CaO19.2183		585	ND	NBD-NBD	ND	Gaur et al., 2005
	CaO19.7332	CaELF1	1097	ND	NBD-NBD	M-RNA export factor	Sturtevant et al., 1998
	CaO19.4152	CaCEF3	978	ND	NBD-NBD	ND	Myers et al., 1992
RLI	CaO19.3034		602	ND	NBD-NBD	ND	Gaur et al., 2005
Others	CaO19.5029		545	ND	NBD-NBD	ND	Gaur et al., 2005
	CaO19.388		276	ND	NBD	ND	Gaur et al., 2005

a *subfamily based on sequence similarity with human ABC transporters*.

b *Number of amino acid residues*.

c *NBD, nucleotide-binding domains; TMS, transmembrane segments*.

*ND, signifies not defined*.

**Figure 2:**
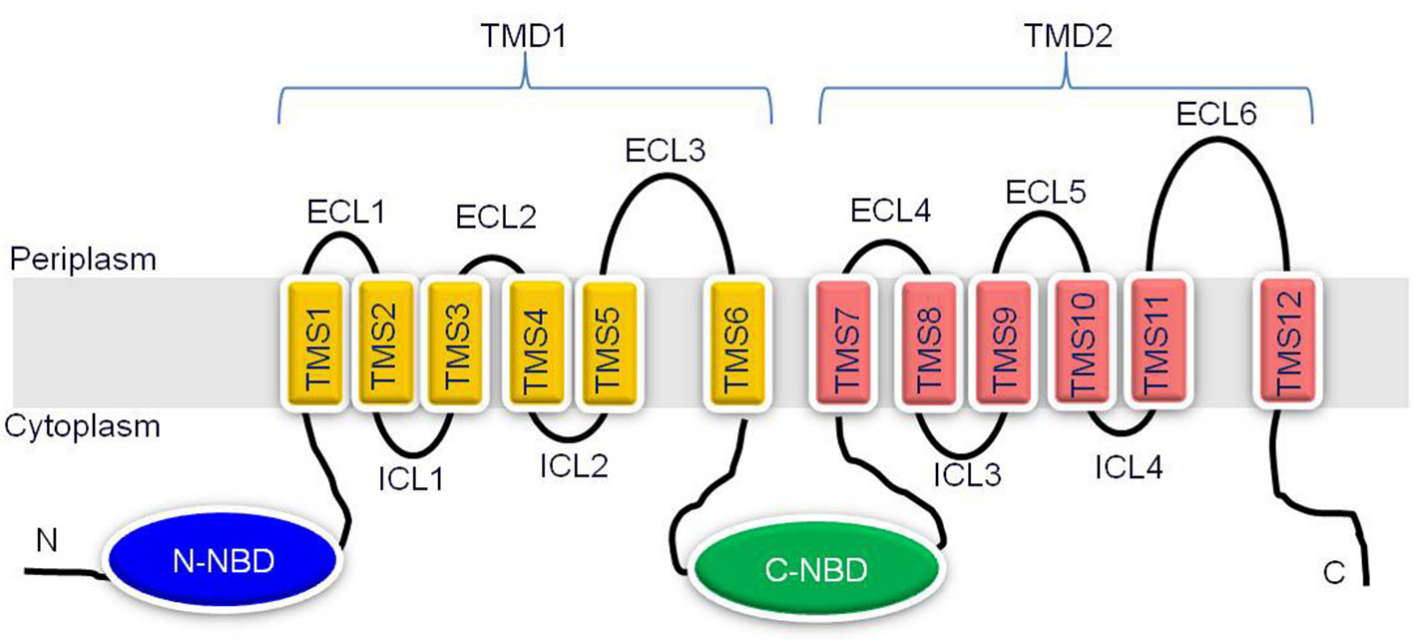
**Schematic representation of the predicted topology of Cdr1p**. Cdr1p is predicted to have two transmembrane domains (TMD1 and TMD2) and two NBDs, (N- and C-terminal NBDs) organized in reverse (NBD-TMS6)_2_ topology. The amino and carboxyl terminals of protein are indicated. Each TMD consists of six TMSs, which are numbered 1–6 in TMD1 and 7–12 in TMD2. Six extracellular loops (ECL1-6) and four intracellular loops (ICL1-4) are indicated.

The Cdr1p represents a major drug transporter of *C. albicans* and an overexpression of its encoding gene is directly associated with an increased drug substrate efflux in azole resistant clinical isolates recovered from patients receiving long term antifungal therapy (Prasad and Goffeau, 2012). Over the years, Cdr1p thus has acquired significant clinical importance and considerable attention is rightly being paid in understanding the structural and functional aspects of this protein. It is expected that such a structural and functional approach could lead to better strategies for designing modulators/inhibitors of this pump enabling combating of antifungal resistance. Cdr1p is an integral plasma membrane protein of 1501 amino acids, with a predicted molecular weight of 169.9 kDa (Prasad and Goffeau, 2012). It is organized as two homologous halves representing reverse topology (NBD-TMD)_2_ as compared to human ABC transporter P-gp. Each half begins with a cytoplasmic hydrophilic nucleotide binding domain (NBD) followed by a highly hydrophobic transmembrane domain (TMD) (Figure 2). Each TMD comprises α-helices of six TMS (transmembrane segments), which creates the substrate promiscuity in the protein (Krishnamurthy et al., 1998a; Puri et al., 2010; Prasad et al., 2011). The NBDs bind and hydrolyse the ATP to drive drug efflux. Conformational changes induced by ATP binding and/hydrolysis are transmitted from the NBDs to the TMDs, which can result in drug translocation, drug efflux or resetting of the reaction cycle.

### Transmembrane domains form promiscuous drug binding site

Despite the overall conservation of the domain architecture of TMDs, their primary sequences are variable among similar transporters. Thus, the extent of conservation is very poor among TMSs of fungal ABC transporters as compared to NBDs which are highly conserved domains (discussed later). The promiscuity toward substrates is a characteristic feature of most ABC drug transporters and hence makes their functionality all the more complex to understand. Therefore, considerable attention is being devoted toward assessment of the structural and functional features in Cdr1p which could explain its diverse substrate specificity spectrum. Understanding the structure activity relationship (SAR) between drug transporter protein and host of xenobiotics they export is expected to improve therapeutic strategies. For this, already there have been concerted efforts to understand the basis of promiscuity of multidrug resistance transporters. Several studies have been undertaken to identify the drug binding site(s) to elucidate the molecular attributes required for substrate/transporter interactions as described below.

The mechanisms of substrate interaction with proteins possessing multiple TMDs are difficult to assess directly because of the absence of crystal structure of these proteins. It is, therefore desirable to take advantage of computational techniques such as three dimensional quantitative structure-activity relationships (3D-QSAR). Identifying the characteristic molecular descriptors is a prerequisite for these computational modeling approaches which have been successfully used in case of human ortholog of the organic cation transporter (hOCT1). The work on Pdr5p of *Saccharomyces cerevisiae*, a close homologous protein of Cdr1p, has offered significant information about the requirement of substrate binding sites. The study by Golin et al. (2000) concluded that hydrophobicity and anion makeup a relatively unimportant factors in determining whether a tri-n-alkyltin compound isa good Pdr5p substrate. However, dissociation of the compound and the molecular size appeared to be an important issue. Although a great deal of similar research has been devoted to the investigation of Cdr1p, still the chemical basis of substrate recognition and transport of these promiscuous transporters of pathogenic *C. albicans* remains speculative. A recent study provided an insight into the physico-chemical basis of substrate specificity of Cdr1p. For this, several compounds of diverse character were randomly selected and screened for their increased toxicity to *S. cerevisiae* strains over-expressing Cdr1p (Puri et al., 2010). On the basis of SAR of substrates and non-substrates, it was observed that, the substrates of Cdr1p had higher hydrophobicity index. The molecular descriptors used in that study further demonstrated that high aromaticity, molecular branching and occurrence of atom centered fragment (ReCHeR) to be the general prerequisites of compounds which are substrates of Cdr1p.

Understanding, how transport protein recognizes such diverse nature of substrate remains a challenge. In an effort to understand the molecular basis of drug recognition, binding and release, several studies have been done to elucidate the location and biophysical properties of the drug-binding sites in Cdr1p. One such study, characterized, three drug binding sites in Cdr1p, one for the binding of rhodamine 6G and azoles such as ketaconazole, miconazole, and itraconazole, second for only fluconazole and third site for iodoarylazido prazosin (IAAP, a photoaffinity analog of the P-gp substrate prazosine, whose binding could be outcompeted by Cdr1p substrate nystatin) (Shukla et al., 2003; Puri et al., 2009). Following extensive site directed mutagenesis of TMS regions of Cdr1p, the role of few TMSs and amino acid residues in in drug transport has been described. For example, the alanine scanning mutagenesis of TMS11 where all the residues were individually replaced with alanine, not only highlighted that Thr1351 of TMS11 of Cdr1p is involved in substrate transport as well as loss of inhibition by FK520, a well known modulator of Cdr1p, but also shed significant light on the role of the entire TMS11. Mutations N1348A, N1359A, L1353A, C1361A, T1351S, and T1355S had no effect on any substrate tested. In contrast, T1355F and F1360A substitution produced unique mutant proteins that drastically lost the ability to confer drug resistance to almost all the tested drugs. But unlike T1351F, they retained the susceptibility to FK520 (Saini et al., 2005). The study showed that all the polar residues of TMS11 are not involved in determining the substrate specificity. The non-conservative substitution (Phe) of Thr resulted in an enhanced susceptibility to drugs whereas more conservative substitution (Ser) had no effect The elimination of the aromatic side chain of Phe1360 decreased the resistance to several fold for all the tested drugs. Together, the study strongly implicated the role of select residues of TMS11 in the mechanism of Cdr1p drug recognition and efflux.

**Figure 3:**
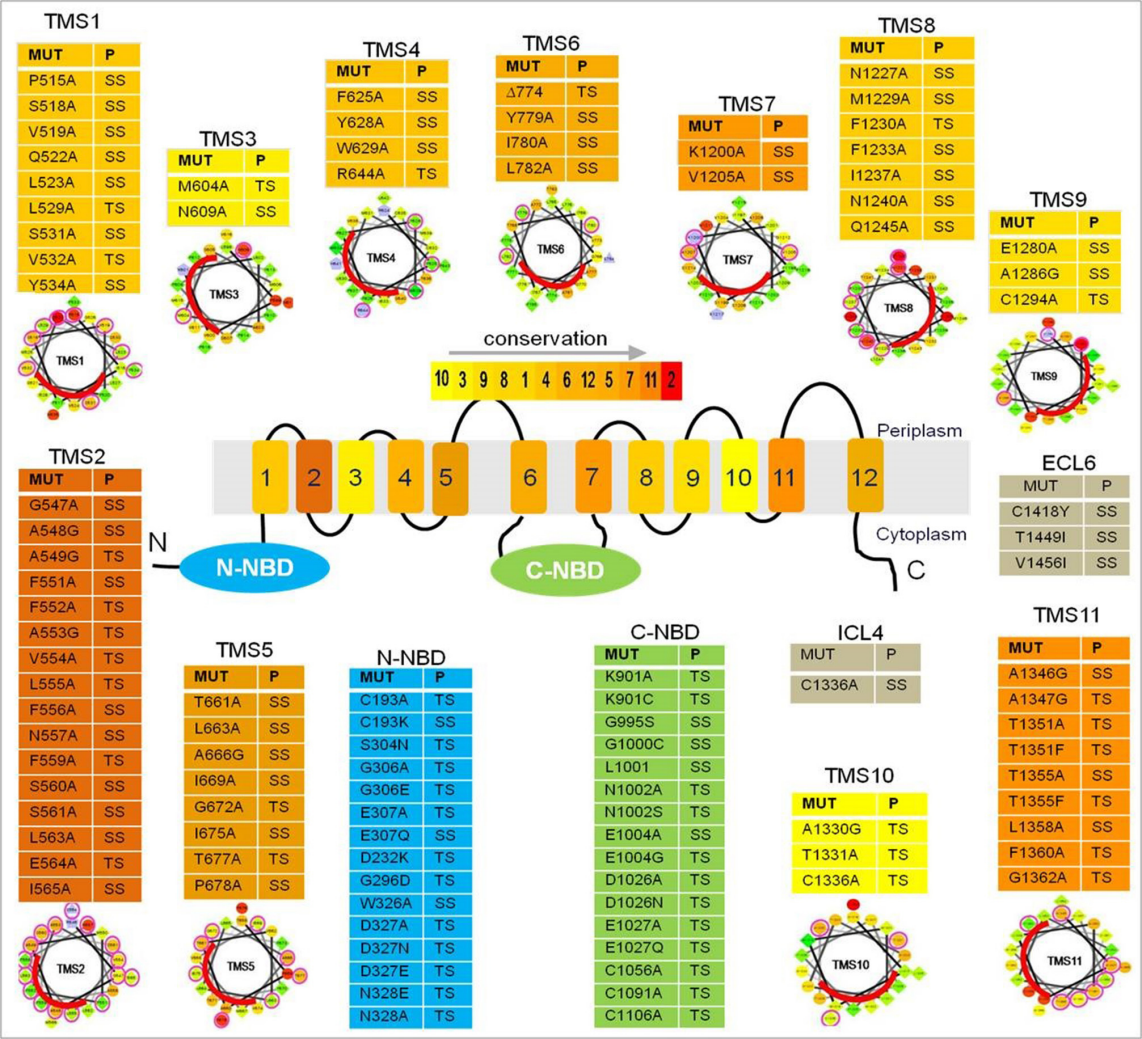
**A list of residues from Cdr1p that upon substitution gave a phenotype**. The color gradient of individual TMS in the central Cdr1p cartoon shows the relative transmembrane conservation based on conservation scores obtained from JALVIEW2.4.0.b2 (Rawal et al., 2013). A red color indicates the highest conservation score while yellow indicates the lowest score. Abbreviations: MUT (mutants), P (phenotype), TS (susceptible to all drugs), SS (selectively susceptible), ECL (extracellular loop), NBD (nucleotide-binding domain), TMS (transmembrane segments). Below each table are the pepwheels showing LIPS (LIPid-facing Surface) residues. Helical wheel projections of each TMS sequence were constructed using http://rzlab.ucr.edu/scripts/wheel/wheel.cgi (Rawal et al., 2013). The sequences are displayed in a helical representation as if looking down the axis of the helix. The mutations that affected drug resistance are circled pink. The red curve on each wheel marks the location of the LIPS surface.

A more recent study probed the nature of drug binding pocket of Cdr1p by performing alanine scanning mutagenesis of the entire primary sequences of the two TMDs, wherein, each of the 252 residues (found in 12 TMSs) replaced with an alanine (Rawal et al., 2013). The study identified several of the replacements to be critical based on the fact that almost all TMSs that, upon mutation, abolished resistance to all drugs tested singly or in combinations (Figure 3) and were predominantly restricted to the non-lipid-facing surface, consistent with close proximity to the substrate-binding pocket. Lipid bilayer-facing surfaces on each TMS of Cdr1p were identified using the empirical scoring function LIPS (LIPid-facing surface), which is based on the lipophilicity and conservation of residues in the helix (Rawal et al., 2013). Residues with high LIPS score (red curves in pepwheels in Figure 3) are expected to face a lipid environment while, the low scoring non-LIPS residues face the substrate-binding site and their substitution is more likely to affect the drug sensitivity of cells over-expressing Cdr1p. Functional data and homology modeling of Cdr1p revealed multiple overlapping mini drug-binding sites within a large centrally located polyspecific drug binding pocket, wherein, all the TMSs, apart from TMS4 and TMS10, interact directly with the drug-binding cavity. The presence of multiple drug binding sites with different requirements for substrate-transporter interaction accounts for the wide variety of substrates known to interact with Cdr1p (Figure 1). The critical residues were found to be predominantly clustered on TMS1, TMS2, and TMS11 on one side of Cdr1 structure and TMS4, TMS5, and TMS8 on another side, while TMS3, TMS6, TMS7, TMS9, and TMS10 contribute relatively fewer such residues. The 3D deduced homology model of Cdr1p also revealed that the amino acid residues that bordered a drug-binding cavity dominated by hydrophobic residues, which is in consistent with Cdr1p having greater affinity toward hydrophobic rather than hydrophilic drugs. Interestingly, no direct correlation was found between the critical residues whose replacement leads to drug susceptibility and the conservation of TMS or the conservation of the residues (Rawal et al., 2013).

### Nucleotide binding domains are the powerhouse of the protein

Although several TMSs associate together to form the drug binding cavity of Cdr1p, binding of drug alone is not sufficient for its transport across the membrane bilayer, it also requires energy from the nucleotide hydrolysis taking place at NBDs to drive the drug extrusion. The NBDs can be regarded as primary sites of generation of energy for powering the efflux of drugs from the cells. Conformational changes induced by ATP binding and/or hydrolysis are transmitted from the NBDs to the TMDs, which can result in drug translocation, drug efflux, or resetting of the reaction cycle (Gupta et al., 2011). In addition to powerhouse of the protein, several residues in NBDs have also been found to alter drug specificity toward many drugs as shown in Figure 3 (Jha et al., 2003, 2004; Shukla et al., 2003; Rai et al., 2005, 2006, 2008; Kumar et al., 2010; Prasad et al., 2012). The typical feature of all yeast ABC transporters of PDR family is that they exhibit high basal ATPase activity and that the most commonly observed feature of drug stimulated ATPase activity component is completely absent from yeast proteins. Cdr1p is also no exception, and displays high basal ATPase activity. Also, the addition of different drugs does not affect the basal activity to any significant level. It implies that, the transporter hydrolyze ATP which is uncoupled from substrate transport (Shukla et al., 2006).

**Figure 4:**
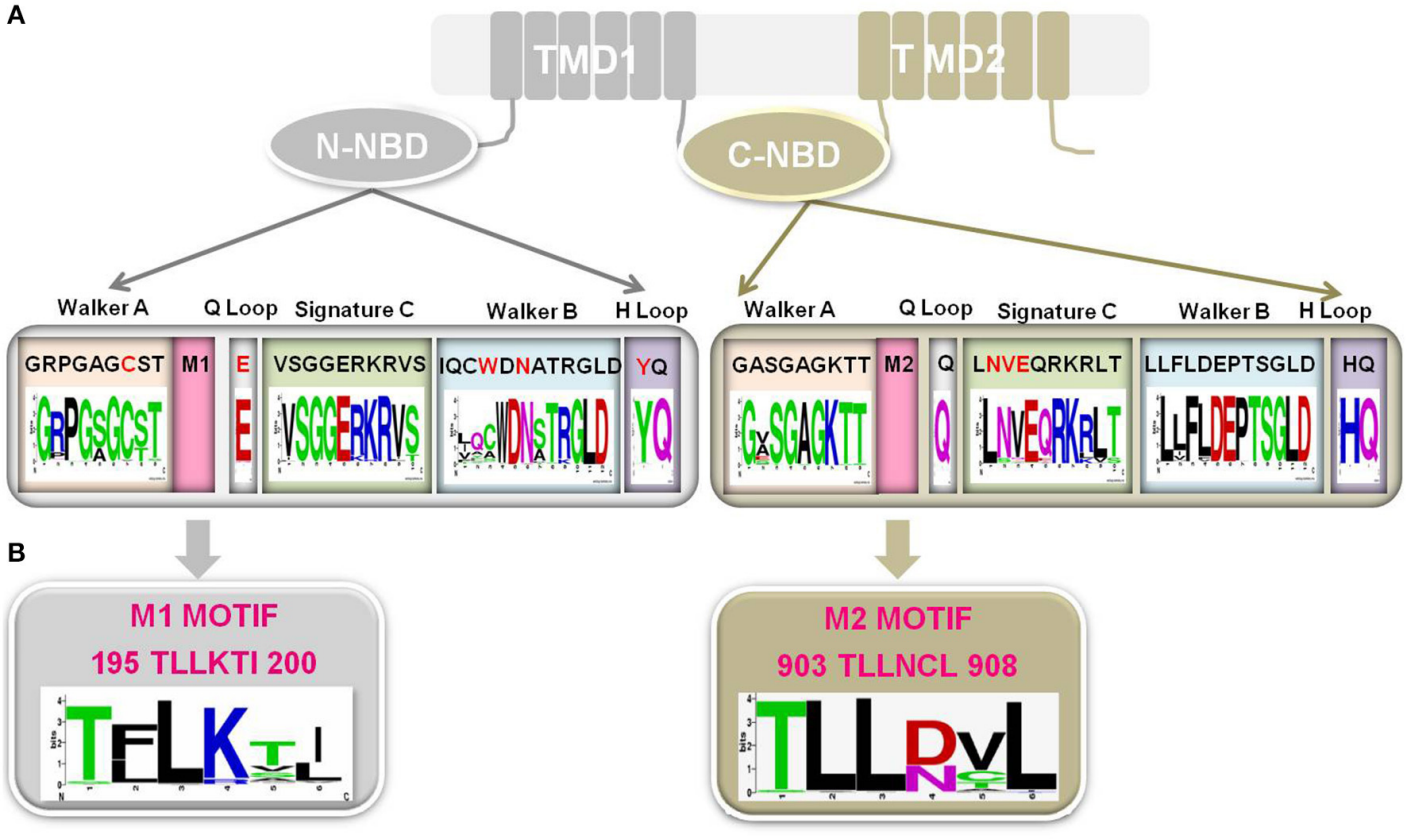
**(A)** Schematic representation of Cdr1p, wherein N-NBD showing degeneracy (red typeface) in conserved Walker A motif, Walker B motif, Q loop and H loop while C-NBD showing degeneracy in signature C. The sequences shown for each motif is from Cdr1p. Sequences of 85 Fungal PDR transporters having topology similar to Cdr1p (Rawal et al., 2013) have been used for the consensus panels below the motif sequences. Sequence conservation of NBD motifs among fungal PDR transporters is individually depicted by Sequence logos, which are a graphical representation of an amino acid multiple sequence alignment. Each logo consists of stacks of symbols, one stack for each position in the sequence. The overall height of the stack indicates the sequence conservation at that position, while the height of symbols within the stack indicates the relative frequency of each amino or nucleic acid at that position. **(B)** A highly conserved PDR subfamily specific motif in Cdr1p which is presenting adjacent to Walker A in both the N-NBD (M1 motif) and C-NBD (M2 motif). Sequence conservation of both the motifs among fungal PDR transporters is individually depicted by Sequence logos.

NBDs are highly conserved in terms of both primary structure and domain architecture as compared to TMSs of TMD1 and TMD2. The NBDs of all ABC transporters, irrespective of their origin and nature of transport substrate, share extensive amino acid sequence identity and the arrangement of typical motifs that are critical for the domain's functionality (Higgins and Linton, 2004). For example, NBDs of ABC transporters have a β-sheet sub-domain containing the typical Walker A and Walker B motifs, as an essential feature of all ATP requiring enzymes (Prasad and Goffeau, 2012), along with an α-helical sub-domain that possesses the conserved ABC signature sequence. A close comparison of the primary sequences of NBDs in fungal PDR proteins reveals that Cdr1p possess unique divergent amino acids in their well conserved motifs. For example, N-terminal NBD (N-NBD) motifs display sequence degeneracy in their Walker A (GRPGAGCST) and Walker B (IQCWDN) motifs, whereas the ABC signature sequence (VSGGERKRVS) remains conserved. In contrast, the Walker A (GASGAGKTT) and Walker B (LLFLD) motifs of the C-terminal NBD (C-NBD) are well conserved and its ABC signature motif (LNVEQRKRLT) is degenerated (Figure 4) (Prasad and Goffeau, 2012). The significance of sequence degeneracy is extensively examined to understand the significance of unique substitutions. The studies so far have established that the unique evolutionary replacements in Cdr1p are functionally indispensable. For example, biochemical analysis of a purified functional N-NBD domain of Cdr1p revealed that the divergent Cys193 of the Walker A motif is essential for ATP hydrolysis (Jha et al., 2003). However, since this observation was an outcome of analyses of an *in vitro* isolated domain, in order to understand the significance of variant Walker A motifs of N-NBD and C-NBD in the context of the full protein, an *in vivo* study was designed, wherein the atypical Cys193 of the Walker A motif of N-NBD (C193K) and conserved Lys901 (K901C) in the Walker A motif of C-NBD were replaced (Jha et al., 2004). The swapping of the cysteine and lysine residues in the Walker A between the two NBDs could not retain the normal ATPase function of the native protein. This result, not only signified the crucial positioning of the Cys193 and Lys901 at their respective domains, but also demonstrated that the swapping of the two residues within the two NBDs is intolerable to the Cdr1p function. Similarly, swapping of entire N-NBD with C-NBD also yielded non-functional protein (Saini et al., 2006). Similarly, exchange of the unique Try326, in the Walker B motif of N-NBD with alanine (W326A) appears to be important for ATP binding and for the accompanying conformational change (Rai et al., 2005). Thus, although the mutant with W326A appears capable of ATP hydrolysis, it does so with a much higher K_M_ value, indicating that the docking of substrate in the binding pocket has been altered by the mutation. The protein, however, appears capable of near-normal drug-transport function in cells expressing the full-length protein carrying the W326A substitution. This implies that the conformational change occurring upon ATP docking cannot by itself be responsible for the cross talk between the NBDs and the TMDs. While the highly conserved Asp327 of N-NBD is known to be the catalytic carboxylate in the context of other ABC transporters, in Cdr1p it does not appear to mediate catalysis via interaction with Mg^2+^, but rather is essential for ATP hydrolysis and acts as a catalytic base (Rai et al., 2006). In a recent study, it has also been suggested that not only certain conserved residues of N-NBD signature sequence (Ser304, Gly306, and Glu307) are important in ATP hydrolysis and drug efflux but also a few divergent residues (Asp1002 and Glu1004) of C-NBD signature motif have evolved to be functionally relevant and are non-interchangeable (Kumar et al., 2010).

The unique substitutions in the N-terminal NBD of fungal transporters do not render the domain non-functional. On the contrary, it is unequivocally shown that the N-terminal NBD of Cdr1p and, by extension those of other fungal transporters, have evolved so as to use their unique substitutions to perform the task of ATP binding and hydrolysis. While it is not yet clear what evolutionary advantage these typical sequence variations might provide to the organisms, it is becoming more and more evident that it has mechanistic implications for the protein. We are yet to understand how the N-terminal NBD works in conjunction with the C-terminal NBD to give rise to a functional drug transporter. Does working in tandem require the ABC Signature sequence of one NBD to participate in ATP binding by the other, as is seen in other ABC transporters? Like in the N-terminal NBDs, the ABC signature sequences of Cdr1p and other fungal transporters too appear to have diverged away from that of other ABC transporters. Whether this is so as to compensate for the substitutions in their N-terminal NBDs or whether they have evolved a new mechanism for coming together for ATP hydrolysis and drug efflux, is a question worth examining.

### A new sub-domain in NBDS of Cdr1 protein

The genome wide inventory of ABC transporters of *C. albicans*, also revealed a new sequence motif characteristic of each subfamily of *C. albicans* ABC proteins. In the transporter subfamilies viz., PDR, MDR, MRP, and ALDp, the new motif occurred in the region between Walker A and Walker B (Figure 4B) whereas in the non-transporter subfamilies viz., EF3 and RLI it occurred next to Walker B (Gaur et al., 2005). Although this new motif can be used to identify sequences from the corresponding subfamily in other organisms, its role in the functioning of ABC proteins remains to be assessed.

### Loops connecting transmembrane segments

The 12 TMSs of yeast ABC transporters are interlinked by six extracellular loops (ECL1-6) on extracellular face and four intracellular loops (ICL1-4) on the cytosolic face of plasma membrane (Figure 2). ECL1, 2, 4, and 5 are generally comparable in length and shorter than other two ECLs (ECL3 and ECL6) which are very long. ICLs have mostly conserved primary sequences compared to ECLs, but are of shorter length (Lamping et al., 2010). The structure and functional studies so far suggest that ECLs act like gates of drug biding pocket, mostly contribute to drug specificity, while ICLs generally serve as intra-domain transmission interface between TMDs and NBDs (Sauna et al., 2008). However, the exact role of ECLs and ICLs in drug transport is not well understood.

#### Extracellular loops

Random mutagenesis of ABC transporter Pdr5p of *S. cerevisiae* led to the identification of several point mutations that caused significant changes in drug specificity which were found to be distributed throughout the length of Pdr5p, particularly, in the extracellular, transmembrane or cytoplasmic regions of the transporter (Egner et al., 1998). For example, Cys1427 in the ECL6 is required for proper protein assembly in the ER membrane and/or the cell surface targeting of Pdr5p. The study also found a double mutation T1460I/V1467I in ECL6 which severely altered the substrates specificity of Pdr5p. Extracellular cysteine residues from ECL6 of Cdr1p from *C. albicans* are highly conserved and critical (Shukla et al., 2003). The ECLs contain conserved sequences that may contribute to pathways used during xenobiotic efflux in fungi, but their form and function is poorly understood (Lamping et al., 2010). Niimi et al have focused ectodomains of Cdr1p by identifying and characterizing a highly specific surface-active inhibitor of the Cdr1p drug efflux pumps that chemosensitizes cells expressing Cdr1p to fluconazole (Niimi et al., 2012). The chemosensitizer was identified, by screening a D-octapeptide combinatorial library for surface-active efflux inhibitors that are not substrates of multidrug efflux pumps. The study identified RC21v3, a potent, stereospecific, surface active, high-affinity inhibitor that interacts with the extracellular surface of Cdr1p. Specific targeting of Cdr1p by RC21v3 was confirmed by isolation of spontaneous chemosensitization-resistant suppressor mutants. Notably, the suppressor mutations introduced a positive charge beside, or within ECL1, 3, 4, and 6 of Cdr1p or an aromatic residue near the extracytoplasmic end of TMS5.

#### Intracellular loops

Fungal ABC transporters belonging to PDR sub-family generally possess peculiar arrangement of ICLs compared to those belonging to MDR type of ABC transporters sub-family. Both the ICLs of each TMD of MDR transporters are large and comparable in size while PDR transporters generally possess one large and one small ICL in each TMD (Figure 2). ICL1 and ICL3 from PDR transporters are comparatively longer in length while ICL 2 and 4 are of shorter length (Lamping et al., 2010). Structure of MDR transporters from bacteria and higher eukaryotes reveals that each TMD contain two ICLs which mediate the communication between NBDs and TMDs through specific coupling helices which interact with Q-loops of NBDs and communicate the conformational changes occurring during ATP hydrolysis to TMDs leading to substrate transport (Ananthaswamy et al., 2010; Lamping et al., 2010).

Recent, biochemical studies have provided evidence of ICLs as communication link between NBDs and TMDs of Pdr5p. For example, drug sensitive phenotype due to point mutations in TMS in Pdr5p is suppressed by mutation in N-NBD possibly by inter-domain communication through ICL1 (Sauna et al., 2008). Point mutation of conserved residue in ICL2 of Pdr5p is also able to restore the drug sensitive phenotype of mutants from Q Loop region of N-NBD (Downes et al., 2013). Similarly A1352M substitution in ICL4 in Pdr5p affects the drug-efflux activity and is shown to be involved in interdomain cross-talk (Guo et al., 2012). Mutations and cysteine cross-linking studies in ICL regions in MDR type ABC transporter Yor1p from *S. cerevisiae* show that ICLs play role in protein folding and membrane localization and in addition also act as a transmission interface between NBDs and TMDs, a situation analogous to ABC proteins of higher eukaryotes (Pagant et al., 2008, 2010). Suppressor mutations, which correct the drug sensitive phenotype in Yor1p, seem to restore the inter-domain communications by the mechanisms different from those observed due to original mutations (Pagant et al., 2008, 2010). Considering the clinical importance of Cdr1p, detailed knowledge of transmission interface and intra-domain communication is essential in designing inhibitors/modulators of the transporter. However, this aspect remains to be examined. The ECLs and ICLs which are interconnecting TMSs are very important with regard to interdomain communication and thus have significant role in drug transport. The work so far has not yet justified their role to fullest and awaits more analysis.

### ABC proteins as phospholipids translocators

Several indirect lines of evidence suggest that fungal ABC transporters such as the *S. cerevisiae* proteins Pdr5p and Yor1p and the *C. albicans* proteins Cdr1p-Cdr4p can translocate fluorescent glycerophospholipid analogs across membranes (Decottignies et al., 1998; Smriti et al., 2002). By employing fluorescent 7-nitrobenz-2-oxa-1, 3-diazol-4-yl (NBD)-tagged phospholipid analogs, it is demonstrated that Cdr1p and its other homologs, Cdr2p and Cdr3p, behave as general phospholipid translocators. Interestingly, *CDR1* and *CDR2*, whose overexpression leads to azole resistance in *C. albicans*, elicit in-to-out transbilayer phospholipid movement (floppase activity), while Cdr3p, which is not involved in drug resistance, carries out-to-in translocation (flippase activity) of phospholipids between the two monolayers of plasma membrane (Smriti et al., 2002). The phospholipid translocation mediated by Cdr1p, Cdr2p, and Cdr3p is distinguishable on the basis of their sensitivities to different inhibitors. The lipid translocation activity of Cdr1p was also confirmed by exploiting a purified Cdr1p reconstitution into membrane vesicles. It is shown that the ATP-driven transporter Cdr1p is capable of translocating different fluorescently modified glycerophospholipids between leaflets of the vesicle membrane. Notwithstanding the role of some of the Cdrps in drug resistance, the study clearly demonstrated that these ABC transporters of *C. albicans* are phospholipid translocators and this function could represent one of the physiological functions of such large family of proteins.

### ABC proteins can transport human steroid hormones

The ability of Pdr5p and Snq2p of *S. cerevisiae* and Cdr1p of *C. albicans* to efflux steroid hormones, like β-estradiol and corticosterone suggests that human steroid hormones could also be the substrates of these drug transporters (Mahe et al., 1996; Krishnamurthy et al., 1998a). Notably, some of the drugs to which Cdr1p overexpression confers resistance, reduce the efflux of β-estradiol and corticosterone probably by mutual competition, thus implying commonality in binding site(s) between steroids and drugs (Krishnamurthy et al., 1998a). Since multidrug transporters of yeast can selectively mediate energy dependent transport of human steroid hormones with high affinity and specificity, it is very likely that these hormones are physiological substrates of these proteins. Ergosterol-the analog of mammalian cholesterol-is the closest molecule to steroids in yeast cells. It is thus quite possible that these multidrug transporters, like human Mdr1p, could be involved in the total sterol homeostasis in the yeast cells (Kontoyiannis, 2000). In this regard, it is also pertinent to mention that progesterone, corticosteroid and estrogen binding proteins in *C. albicans* have already been identified (Das and Datta, 1985). The interaction of ketoconazole with corticosteroid binding protein has also been observed (Stover et al., 1983). Additionally, the upregulation of *CDR1* transcription by steroid hormones and recent identification of steroid responsive region in its promoter do strongly point to a possible link between the steroid response cascade and multidrug resistance in *C. albicans* (Krishnamurthy et al., 1998b).

## MFS proteins in *Candida*

The major facilitator superfamily (MFS) proteins are another class of major drug transporters which are involved in the development of multidrug resistance in yeasts. MFS transporters, like ABC transporters, belong to a large superfamily of membrane proteins from bacteria to higher eukaryotes which include symporters, antiporters, and uniporters of various substrates (Marger and Saier, 1993) (Figure 1). The MFS proteins are secondary active transporters which utilize the electrochemical gradient of protons to drive transport of various substrates against concentration gradient across the membrane (Ben-Yaacov et al., 1994). The genome wide inventory revealed that *C. albicans* genome has 95 putative MFS proteins that clustered into 17 families (Gaur et al., 2008). Out of all MFS proteins, only CaMdr1p is known to extrude drugs, where its overexpression has been linked to azole resistance (Kohli et al., 2001). CaMdr1p over expression is linked to an emergence of resistance to several unrelated drugs e.g., fluconazole, 4-nitroquinoline–N-oxide, cycloheximide, benomyl, methotrexate and cerulenin (Figure 1) (Ben-Yaacov et al., 1994; Gupta et al., 1998; Prasad and Kapoor, 2005). The expression of *CaMDR1* in *C. albicans* gets enhanced by benomyl, methotrexate and several other unrelated drugs, and was more pronounced in some of the azole resistant clinical isolates (Gupta et al., 1998). This confirms that over-expression of *MDR1* gene directly relates to azole resistance in clinical isolates of *C. albicans* (Gupta et al., 1998). Another MFS transporter, FLU1 which is implicated in mycophenolic acid resistance is also characterized. However, no clinical relevance of this drug transporter has been reported so far.

**Figure 5:**
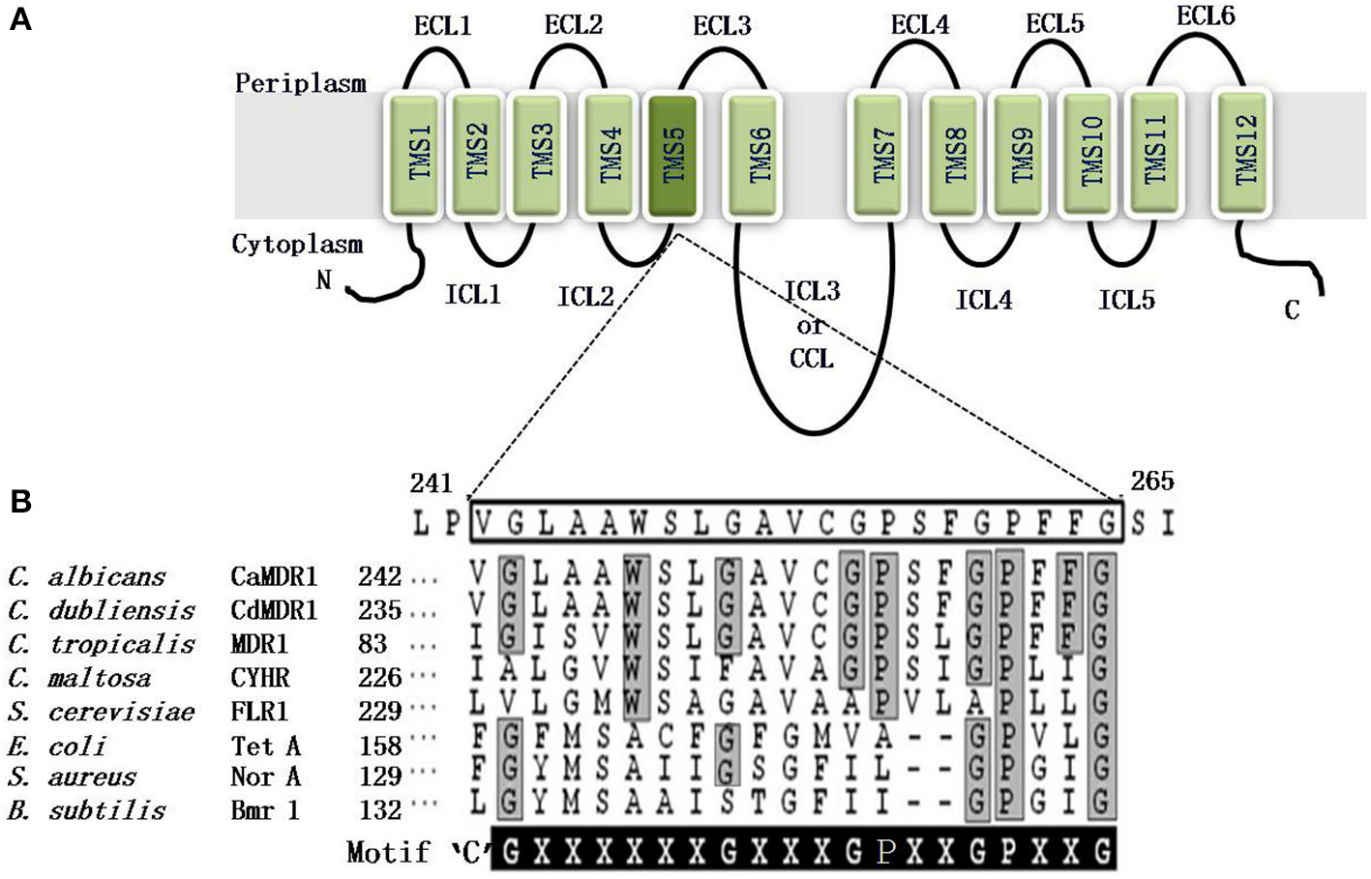
**(A)** Predicted topology of the CaMdr1p with 12 transmembrane segments. The amino and carboxyl terminals of protein are indicated. Six extracellular loops (ECL1-6) and five intracellular loops (ICL1-5) are indicated. Two homologous halves are connected by a central cytoplasmic loop (CCL) or ICL3. The TMS5 is colored dark and magnified to show the amino acid residues of TMS5. **(B)** Alignment of the protein sequences of the *C. albicans* antiporter CaMdr1p TMS5 with the other fungal and bacterial drug antiporters, showing the presence of the unique and conserved antiporter motif or motif C. The amino acid sequence of TMS5 of CaMdr1p is boxed. The sequence of the antiporter motif is written for comparison, where X can be any amino acid. Residues conserved in all the MFS transporters that are part of the motif are highlighted in gray, whereas residues conserved only in fungal MFS that were found critical for the activity are highlighted in black.

MFS consists of at least six separate families (Paulsen et al., 1996). On the basis of hydropathy and phylogenetic analysis the drug efflux MFS proteins are divided into two distinct types; first, Drug: H+ Antiporter-1 (DHA1), consisting of 12 TMSs and the second is called Drug: H+ Antiporter-2 (DHA2) that contains 14 TMSs. The CaMdr1p is a 564 amino acid protein of *C. albicans* that belongs to the DHA1 (drug–proton antiporter) family, which effluxes drugs in exchange with one or more H+ with a substrate molecule (Paulsen and Skurray, 1993). It is characterized by two structural units of six TMS-α-helical segments, linked by a cytoplasmic loop. Six TMSs are interconnected by ECLs and ICLs (Figure 5).

### Structure and function studies in CaMdr1p

In contrast to ABC drug transporter, the structure-function relationship of MFS proteins has not been analyzed in detail. There are, however, some reports to suggest that the N-terminal halves of different major facilitator families share greater similarities than their C-terminal halves, which suggests that C-terminal regions are involved in substrate recognition, and N-terminal regions are involved in proton translocation (Saier and Reizer, 1991). Additionally, the MFS drug antiporter proteins possess many conserved residues scattered throughout the length of the protein, for example, motifs A and B are conserved throughout the MFS, while motif “C” is found only in 12 and 14-TMS subfamilies (Paulsen et al., 1996).

**Figure 6:**
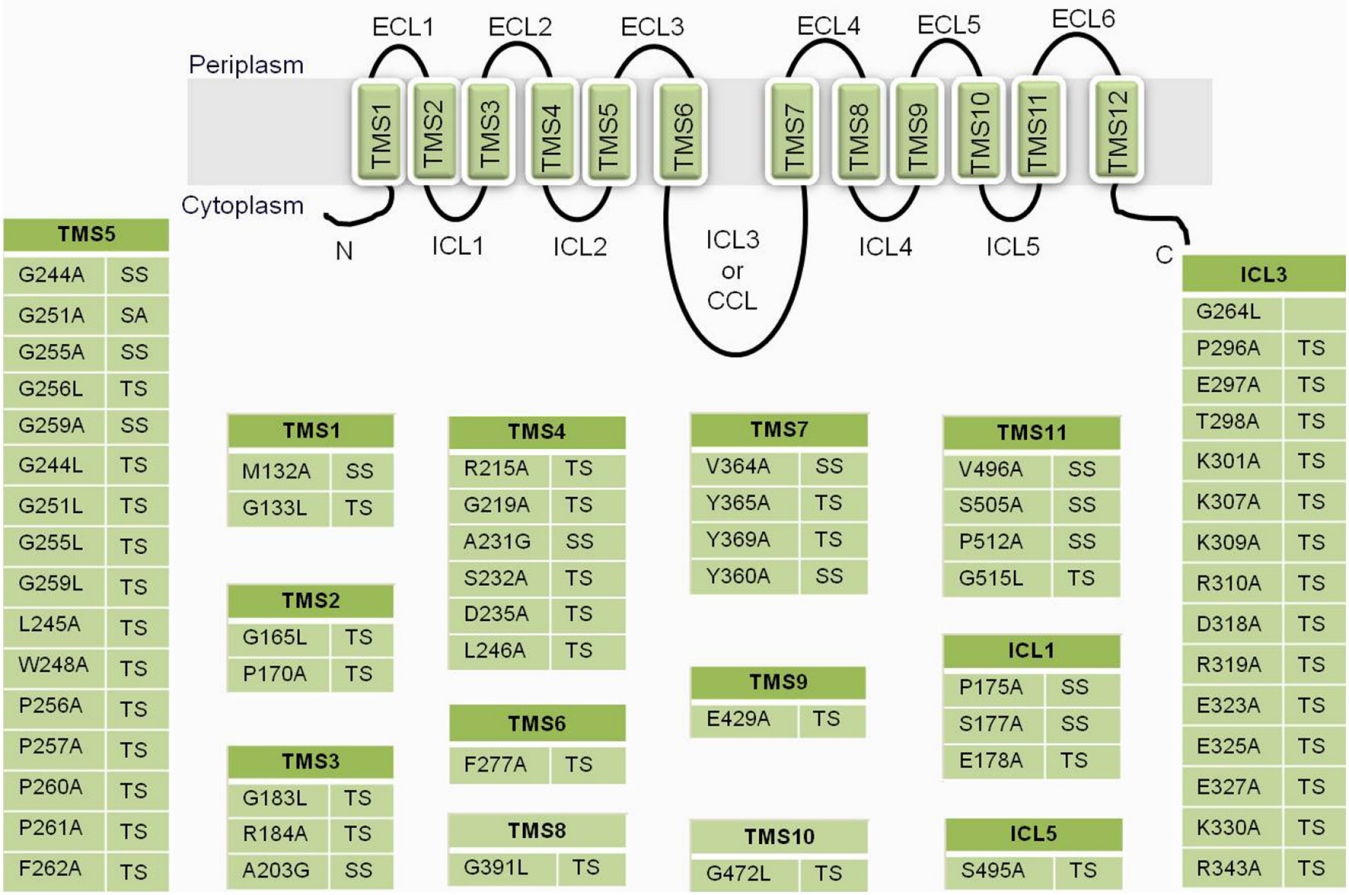
**A list of residues from CaMdr1p that upon substitution gave a phenotype**. TS, susceptible to all drugs; SS, selectively susceptible; ECL, extracellular loop; TMS, transmembrane segments; ICL, intracellular loops; CCL, central cytoplasmic loop.

The bacterial MFS drug/H+antiporters, which have a unique antiporter motif, also called motif “C” [G(X8)G(X3)GP(X2)GG], is necessary for the drug/H+antiporter activity (Ginn et al., 2000). Independent of the substrate promiscuity of the antiporter, the antiporter motif in the predicted TMS5 is conserved in all of the functionally related subgroups in bacteria and plants. However, based on sequence homology, CaMdr1p is a putative proton antiporter of *C. albicans*, with an antiporter motif in TMS5 [G(×6)G(×3)GP(×2)GP(×2)G]) as depicted in Figure 5. Structural and functional analyses of CaMdr1p have indicated that amino acid residues located in TMS5 are critical for drug/proton transport activity (Figure 6). The clustering of several important residues including those of motif “C” which participate in substrate specificity has been observed on one face of the helix (Pasrija et al., 2007).

### Rational mutational analysis

Site-directed mutational strategies rely on conservation of residues in a multiple sequence alignment (MSA). The conservation of a residue is calculated from the amino acid frequency distribution in the corresponding column of a MSA. However, the physicochemical conservation is not necessarily responsible for a protein's structure and function but could reflect a more general function such as membrane localization. Thus, conservation alone is not sufficient to distinguish between residues responsible for the protein function and membrane localization. Membrane proteins differ from soluble proteins because of their inter-TM hydrophilic and TM hydrophobic propensities, which have allowed the development of efficient membrane protein TM prediction methods and of membrane protein specific substitution matrices. Recent study employed a membrane environment based computational approach to predict the functionally critical residues of CaMdr1p. For this, DHA1 (antiporters) family was contrasted with sugar porter family (SP) (symporters) to identify residues selectively conserved in DHA1 family vis-à-vis the SP family. The residues with highest cumulative relative entropy (CRE) were short-listed for analysis and were predicted to impart functional specificity to the DHA1 members. The functional relevance of these high-CRE scoring residues of CaMdr1p was evaluated by employing site directed mutagenesis. However, only those positions were mutated to alanine or leucine wherein the CaMdr1p residue matched with the most frequent amino acid at a particular alignment position of DHA1members. Most of the mutant variants displayed complete drug susceptibility while few were selectively susceptible to tested drugs (Figure 6). The enhanced susceptibility of these mutant variants was corroborated with abrogated efflux of substrates rather than poor expression or surface localization. Since the prediction was based on entire DHA1 family, this strategy, not only identified critical residues of CaMdr1p, but also accurately predicted and validated family-wide-function residues for this entire subfamily of antiporters (Kapoor et al., 2010).

### Interdomain communication

Several members of MFS proteins including fungal drug-proton antiporter (DHA1) family (Saier et al., 2006) possess a central cytoplasmic loop (CCL), large enough to form a cytosolic domain (Weinglass and Kaback, 2000; Monden et al., 2001; Xu et al., 2010). The helices in this domain are rich in non-polar amino acids, which impart considerable amphipathicity to the loop for establishing contacts with the plasma membrane (Xu et al., 2010). The CCL connecting TMDs plays an important functional role in several MFS proteins. For example, the CCL of *LacY* (lactose permease of *Escherichia coli*) transporter (Weinglass and Kaback, 2000), Dtr1p (di-tyrosine transporter) of *S. cerevisiae* (Morishita and Engebrecht, 2008), human RFC1 (reduced folate carrier) (Sadlish et al., 2002) and hPEPT1 (intestinal di-/tri-peptide transporter) (Xu et al., 2010) are necessary for either their efficient membrane insertion, proper maturation, expression or function. In human GLUT1 (Glucose transporter 1), the conformational changes within TMHs induced by the initiation of efflux activity is shown to be communicated through CCL of the protein (Monden et al., 2001).

In a recent study, to evaluate the role of ICL3 or CCL of Mdr1 protein of *Candida*, a mutational strategy was employed to the entire stretch of amino acid residues of the loop (Mandal et al., 2012). The two sets of mutant variants of CaMdr1p were constructed. One group of variants included progressive nine deletants of different stretches of 6–7 residues and the other group included variants where respective deletions were restored with hexa- or hepta-alanine. The data clearly showed that most of the deletion and substitution of ICL3 sequence stretches resulted in non-functional protein variants of CaMdr1p that totally eliminated their ability to confer multidrug resistance and substrate efflux. Additionally, the progressive deletion and its restoration lead to change in protein conformation and surface charge distribution that was evident from the accessibility of protease digestion of variants resulting in multiple tryptic fragments. A close examination of the CCL sequence reveals that the N-terminal half of the loop mainly contains positively charged amino acid residues, whereas the C-terminal half contains negatively charged amino acids. The replacement of most of the positively charged side-chain mutants in N-terminal half of the loop with alanine completely eliminated their ability to tolerate drugs (Figure 6). Together, the study on ICL3 of CaMdr1p not only highlights the critical role of CCL in fungal MFS belonging to DHA1 family in drug transport but also shows that the large inter-domain central ICL3 connecting the two TMDs is an essential structural element of protein.

## Concluding remarks

Both ABC and MFS transporters belong to two big super families of proteins and several of them have also evolved as drug transporters. Interestingly, though they differ in terms of energy coupling to drug transport, (ABC proteins power drug export by directly hydrolyzing ATP, the MFS transporters employ electrochemical gradient of protons to active drug export), the promiscuity with regard to their ability to transport variety of structurally unrelated compounds, of the two families of transporters is most common striking feature. This promiscuity of such proteins makes it a big challenge in dissecting their structure and function. From the recent studies, it is evident that lot of efforts are devoted in resolving the mechanisms of drug transport. It is confirmed that TMSs of these transporters harbors drug binding sites and critical amino acid residues align and make up a large drug binding pocket which can accommodate diverse compounds to export. The challenge lies in dissecting participation of important residues responsible for single or select drug transport. Unlike MFS transporetrs, several ABC drug transporters are phospholipid translocators which may reflect their normal physiological function. But understanding how phospholipids and drug are transported by these proteins is a challenge. Nonetheless, resolving such issues will pave the way in rationally designing inhibitors/modulators for making drug exporters transport incompetent so that they could chemosensitize the drug resistant cells.

## Author contributions

Rajendra Prasad: drafting the work and revising it critically for important intellectual content; and final approval of the version to be published. Manpreet K. Rawal: contributed in drafting the work.

### Conflict of interest statement

The authors declare that the research was conducted in the absence of any commercial or financial relationships that could be construed as a potential conflict of interest.

## Abbreviations

P-gp: P-glycoprotein
ABC: ATP binding cassette
MFS: major facilitator superfamily
MDR: multidrug resistance
PDR: pleiotropic drug resistance
NBD: nucleotide binding domain
TMD: transmembrane domain
TMS: transmembrane segments
ECLs: extracellular loops
ICL: intracellular loops
CCL: central cytoplasmic loop.
